# Sustained pharyngeal inflation in infant airway—Flexible bronchoscopy measurements

**DOI:** 10.1371/journal.pone.0294029

**Published:** 2023-11-22

**Authors:** Christina Soong, Yu-Sheng Lee, Chien-Heng Lin, Chieh-Ho Chen, Wen-Jue Soong

**Affiliations:** 1 Institute of Biomedical Engineering, College of Medicine and College of Engineering, National Taiwan University, Taipei, Taiwan; 2 Department of Physical Medicine and Rehabilitation, National Taiwan University Hospital, Taipei, Taiwan; 3 Department of Pediatrics, School of Medicine, National Yang-Ming Chiao Tung University, Taipei, Taiwan; 4 Division of Pediatric Pulmonology, Children’s Hospital, China Medical University, Taichung, Taiwan; 5 Department of Pediatrics, Tri-Service General Hospital, National Defense Medical Center, Taipei, Taiwan; Universiti Malaya Fakulti Perubatan: University of Malaya Faculty of Medicine, MALAYSIA

## Abstract

Sustained pharyngeal inflation (SPI) with pharyngeal oxygen flow and nasal closure (PhO_2_-NC) technique create positive inflation pressure in the airway. This study measured the peak inflation pressure (PIP) levels and image changes with SPI-assisted flexible bronchoscopy (SPI-FB) and compared the effects in the pharyngeal space and mid-tracheal lumen. This prospective study enrolled 20 participants aged 6 months to 3 years. Each participant underwent sequential SPI-FB of four different durations (0, 1s, 3s, and 5s) for three cycles. We used a 3.8 mm OD flexible bronchoscope to measure and analyze PIP levels, images, and lumen dimension scores. A total of 480 data were collected. The mean (SD) age and body weight were 12.0 (11.5) months and 7.8 (7.5) kg, respectively. The mean (IQR) PIPs were 4.2 (2.0), 18.5 (6.1), 30.6 (13.5), and 46.1 (25.0) cmH_2_O in the pharynx and 5.0 (1.6), 17.5 (6.5), 28.0 (12.3), 46.0 (28.5) cmH_2_O in the mid-trachea at SPI durations of 0, 1s, 3s, and 5s, respectively. The PIP levels had a positive correlation (p <0.001) with different SPI durations in both pharynx and trachea, and were nearly identical (*p* = 0.695, 0.787, and 0.725 at 1s, 3s, and 5s, respectively) at the same duration except the 0 s (*p* = 0.015). Lumen dimension scores also significantly increased with increasing SPI durations (*p* <0.05) in both locations. The identified lesions significantly increased as PIP levels increased (*p* <0.001). Conclusion: SPI-FB using PhO_2_-NC with durations up to 3s is safe and informative technique that provides controllable PIP, dilates airway lumens, and benefits lesion detection in the pharyngeal space and mid-tracheal lumen.

## Introduction

Traditionally, flexible bronchoscopy (FB) in children frequently complicated with hypoxia and hypoventilation, therefore, that are recommended using noninvasive ventilation (NIV) support include artificial devices of Ambu bag, mask, nasal prongs, laryngeal mask airway, or respiratory machine to maintain adequate oxygenation and ventilation [1–3]. However, if structural or functional airway anomalies are suspected, patients should be allowed breathe with their own natural airway, without distortions by any artificial devices, and performing the whole airway evaluation. The flow of air into the lungs requires a pressure gradient between the atmosphere, pharynx and the alveoli. Intermittent sustained pharyngeal inflation (SPI) is one technique of NIV which creates the changes of pressure in pharyngeal space that result in lung ventilation. In recent years, the SPI has gradually been popular, particularly when application in neonatal resuscitation and the prevention of bronchopulmonary dysplasia [4–7].

Our team has previously reported clinical application of a novel NIV technique, pharyngeal oxygen with optional nose-closure and abdomen compression (PhO_2_-NC-AC), for several years [8–11]. This technique has many benefits of simple and practical in needs no artificial airways or ventilation devices. It has proven feasibility, safety, and effectiveness in providing adequate oxygenation and positive pressure ventilation (PPV) in diagnostic and therapeutic FB of whole approachable airways in pediatric patients [8–17], even in those with severe compromised airway and high-risk cardiopulmonary status. Parts component of this NIV technique, pharyngeal oxygen with nasal closure (PhO_2_-NC), can be carried out as a SPI. In our previous study [18], the SPI-assisted flexible bronchoscopy (SPI-FB) [S1 Video] demonstrated a positive correlation between the durations of SPI and the levels of peak inflation pressure (PIP), lumen dimensions, and more detail visualization in the pharyngolaryngeal space. However, no study has explored the effects of different SPI durations on the upper and lower airway lumens. We hypothesize that this SPI technique may also affect both upper and lower airway lumens similarly and simultaneously.

The aims of this study are measured and compared the effects of different SPI durations on PIP levels, airway lumen images, and identifying lesions in children’s pharyngolaryngeal space and tracheal lumen.

## Methods

This prospective study enrolled 20 infants and young children from January 2021 to December 2021. The inclusion criteria are:

1. Age between 6 months and 3 years.
2. Scheduled for a FB examination due to a suspected airway lesion.

The exclusion criteria were subjects with 1) bilateral nasal stenosis that inhibited entry of a 4.0 mm out diameter flexible bronchoscope, 2) craniofacial dysmorphisms, 3) obvious cardiopulmonary compromise, which could not tolerate this study, and 4) guardians had not signed a writing informed consent for this SPI-FB study. In order to minimize technical bias, experienced bronchoscopists carried out all the SPI-FB procedures. The hospital’s Institutional Review Board and ethic committee (Research Ethics Committee, China Medical University & Hospital, Taichung, Taiwan approved this study (DMR-110-062 and CMUH109-REC1-194, respectively). Patient confidentiality was maintained in accordance with the Health Insurance Portability and Accountability Act guidelines.

### Preparation: Sedation and respiratory support

Enrolled candidates followed a standard preparation for their scheduled FB as usual. Vital signs, including blood pressure, heart rate, respiratory rate, and oxygen saturation, were monitored continuously. Intravenous injection of atropine sulfate 0.01mg/kg was given 20 min before the examination. Procedural sedative medication with intravenous midazolam 0.1–0.2mg/kg and ketamine 1–2mg/kg were administrated to keep the subject motionless while maintaining spontaneous breathing as possible. Their original respiratory support devices such as a nasal cannula, nasal prongs, masks, endotracheal tube, and ventilator, were removed. Topical anesthesia with 2% lidocaine solution, 0.5–1.0 ml/kg (maximum 10 ml), was instilled into the subjects’ nostrils and tracheal lumen using a syringe catheter.

### NIV using PhO_2_-NC-AC support for scheduled FB

**Fig 1:**
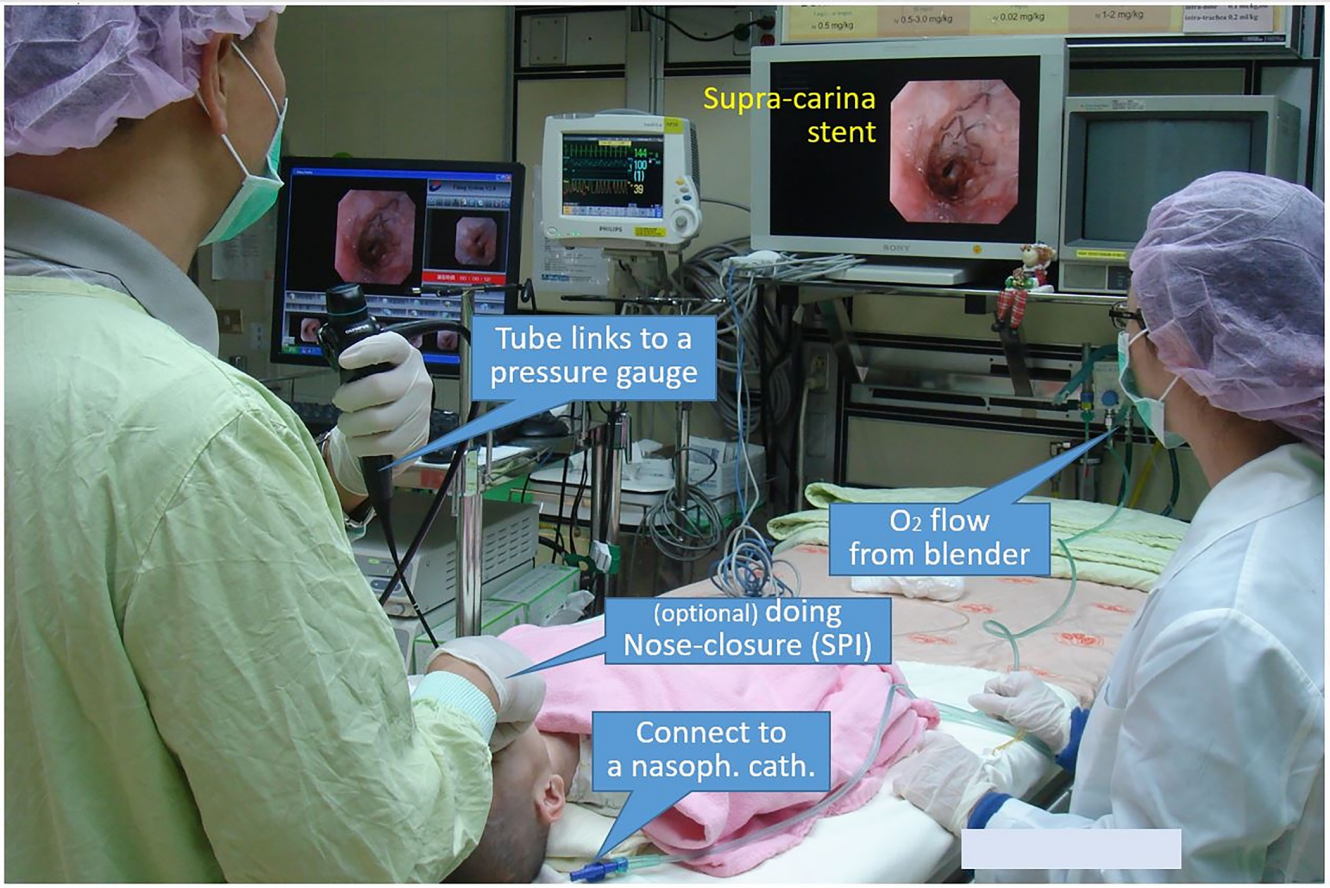
Gross picture of setting circuit in sustained pharyngeal inflation assist flexible bronchoscopy. Warm and humidified oxygen flow delivers from blender, through a tube, and links to a nasopharyngeal catheter to the pharynx; bronchoscope’s inner channel connects to a pressure gauge.

A warm, humidified, and continuous pure oxygen flow was delivered (via Fisher & Paykel MR 810) at a rate of 1.0 L/Kg/min (maximum 10.0 L/min) (Fig 1), through a 10-French suction catheter inserted via the right nostril as a nasopharyngeal oxygen catheter. The operator’s left-hand held the flexible bronchoscope.

**Fig 2:**
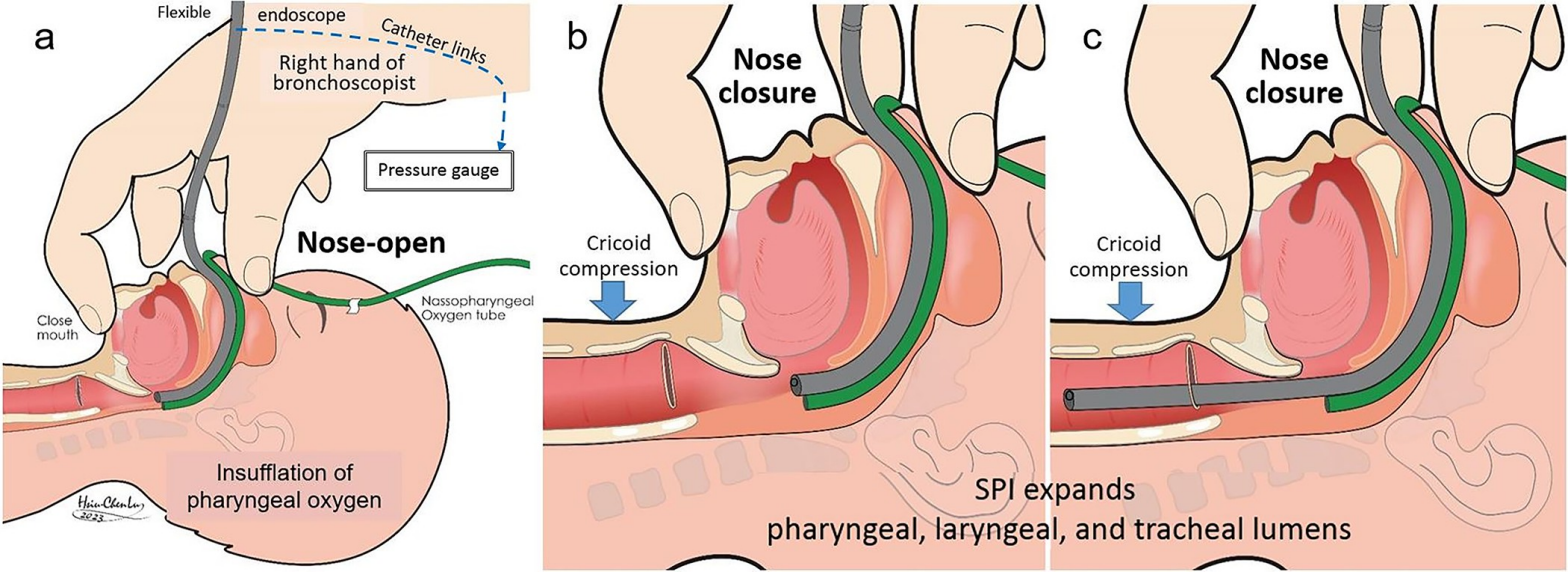
Technique of flexible endoscopy with sustain pharyngeal inflation support. (a) Nose-open presents with narrow airway space; sustained inflations (nose-close) expand both upper and lower airway lumens: partial enlarged images, endoscope tip in (b) the pharynx and in (c) the trachea.

Operator’s right hand closed around the subject’s oronasal area (Fig 2A). The pharyngolaryngeal space was then filled with warmed and humidified oxygen (PhO_2_). To provide inspiration, the operator performed nasal–closure (NC) by pinching the nose with the right-hand thumb and middle finger while closing the mouth by hooking the mandible with the index finger. During the FB, the tip of endoscope advance from the pharynx (Fig 2B) to the mid-tracheal lumen (Fig 2C). For expiration, NC was released, and abdominal–compression (AC) with the right hand pressed over the umbilical area was done simultaneously. Both actions of the NC and the AC were optionally performed at a rate of 0 to 5 cycles/min, as needed. This supportive NIV using PhO_2_-NC-AC was accomplished throughout the FB examination.

### SPI-FB study

**Fig 3:**
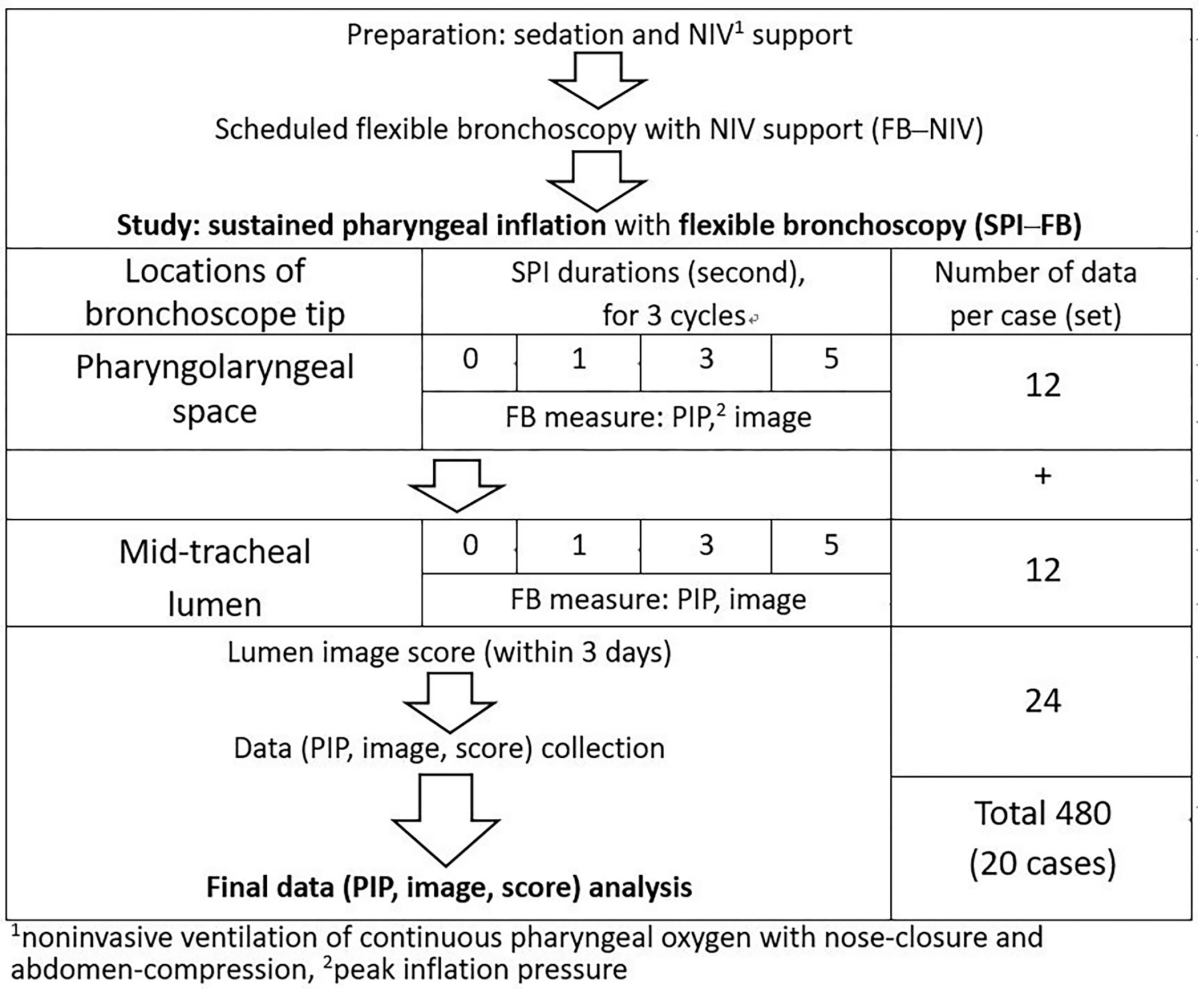
Algorithm of study. Sustained pharyngeal inflation–flexible bronchoscopy (SPI–FB) measurements in each enrolled infant (total 20 infants).

A working length of 25 cm flexible bronchoscope (HYF-V, Olympus), with an outside diameter of 3.8 mm and an inner channel of 1.2 mm, was used for this study. A small pressure-transmitting catheter connected the inner channel to a real-time pressure gauge (GB30, Galemed, Taiwan). The study algorithm was carried out as shown in Fig 3.

### Modes of SPI, PIP and image measurement

The flexible bronchoscope was inserted via the left nostril and gradually advanced from pharynx, larynx to trachea. Throughout the course, SPI with NC maneuver was performed at two locations, the pharyngeal space and the mid-tracheal lumen. PIPs and images were taken simultaneously at the end of four different NC durations (0, 1, 3, and 5 s) at the same locations. The pressure gauge showed PIP levels, while the bronchoscope captured images. Between each NC, a 10-s open-nose period allowed for deflation and spontaneous breathing. Each enrolled subject took three cycles of these measurements in the pharyngolaryngeal space and mid-tracheal lumen.

At the end of the SPI-FB study, AC and gastric suctioning were performed on each subject to prevent abdominal distension. Chest radiograph was routinely checked to identify any possible sequelae, such as air leaks or other associated lesions.

### Lumen dimension scoring

For each participant, 24 images were collected and sent for objective evaluation within 3 days. Image dimension scores were judged on a 5-point scale (1: severely collapsed, 2: collapsed, 3: average, 4: expanded, 5: significantly expanded) by five qualified and independent pediatric pulmonologists unaware of the image sources. Individual scores were analyzed regarding the various SPI durations and PIP levels.

### Statistical analysis

PIP levels and image scores were analyzed using One-way ANOVA with MedCalc statistical software (version 19.3.1, MedCalc Software Ltd, Acacialaan 22, 8400 Ostend, Belgium). The PIP level at 0-s of NC served as the baseline. Analyses for the numbers of identified lesions between different SPI durations were performed using chi-square and Bonferroni post hoc tests. Statistical differences were considered significant at *p* < 0.05.

## Results

The mean (SD) age of the 20 enrolled infants at the time of SPI-FB was 12.0 (11.5) months old, and the mean (SD) body weight was 7.8 (7.5) kg. The duration of the entire procedure of this SPI-FB study, from introduction of the endoscope to withdrawal, was completed with the mean (SD) time of 8.7 (2.1) min. A total of 480 sets of data, composed of PIP levels, images, and lumen dimension scores were gathered and analyzed. There was neither study-related complications nor adverse events, such as airway bleeding, air leak, pneumothorax, desaturation (SpO_2_ less than 90%), or bradycardia (heart rate less than 100 beat/min) for more than 20 s.

Table 1 summarizes and analyses the measured data at different SPI durations. A positive and significant (*p* <0.001) correlation existed between the measured PIPs and the SPI durations in both the pharyngeal space and mid-tracheal lumens. The same results (*p* <0.01) also shown in the lumen dimension score. In addition, the number of identified lesions significantly (*p* = 0.001) increased with increasing duration of SPI. Compared to the control group at 0-s, SPI durations of 3-s (*p* = 0.005) and 5-s (*p* <0.001) could identify more lesions. Among them, the 5-s SPI produced the highest PIPs, lumen dimension scores, and the greatest number of detected lesions.

**Table 1:** Summary and comparison of sustained pharyngeal inflation (SPI) study data.

SPI study	Variables in different SPI durations				*P*
SPI duration (seconds)	0 (control)	1	3	5	
**PIP level, median (IQR)**^2^, **cmH**_**2**_**O**					
Pharyngeal space	4.2 (2.0)	18.5 (6.1)	30.6 (13.5)	46.1 (25.0)	<0.001
Mid-tracheal lumen	5.0 (1.6)	17.5 (6.5)	28.0 (12.3)	46.0 (28.5)	<0.001
**Lumen dimension score** ^3^					
Pharyngeal space	1.1 (0.2)	2.4 (0.3)	3.6 (0.4)	4.7 (0.2)	<0.01
Mid-tracheal lumen	1.2 (0.3)	2.5 (0.4)	3.7 (0.4)	4.6 (0.3)	<0.01
**Identified lesion**					
Pharyngeal space					
Pharyngomalacia	1	2	2	2	
Vallecular cyst with laryngomalacia				1	
Laryngomalacia	1	1	1	2^4^	
Laryngeal cleft			1	1	
Vocal cords palsy		1	1	1	
Tracheobronchial lumen					
Tracheomalacia	2	2	2	2	
Tracheoesophageal fistula		2	2	2	
Carina malacia			2	2	
Total lesion number (%)	4 (30.7)	8 (61.5)	11 (84.6)	13 (100)	0.001
^*5*^ *p*		0.116	0.005	<0.001	

Measured PIP,^1^ lumen dimension score and number of identified lesions in pharyngeal space and mid-tracheal lumen by flexible bronchoscopy with four different durations of SPI support.

^1^Peak inflation pressure; ^2^interquartile range, ^3^five degree of score, 1 to 5: severe collapsed, collapsed, average, expanded, much expanded, respectively; ^4^one lesion detects below the vallecular cyst, ^5^SPI duration vs. control, chi-square with Bonferroni post-hoc test.

**Fig 4:**
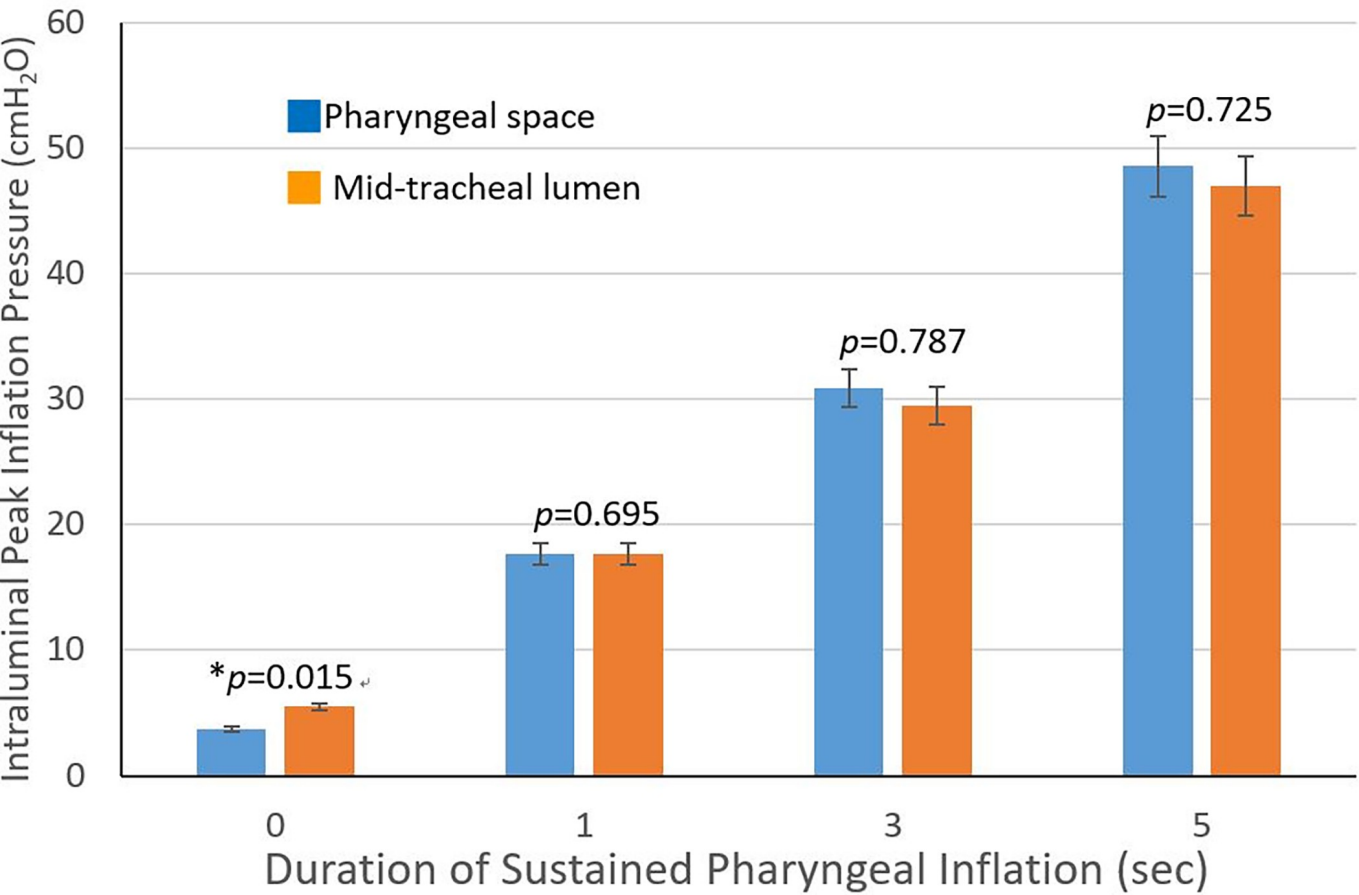
The measured peak inflation pressure (PIP) in pharynx and mid-trachea. The PIP levels show significant positive correlation with durations of sustained pharyngeal inflation (SPI). Nearly identical PIP levels between the pharynx and mid-trachea, except in the 0 second.

In addition, these PIP levels were nearly identical, as presented in Fig 4, in both sites of pharyngeal space and mid-tracheal lumen at each SPI duration, except the control group with no SPI (*p* < 0.05). The *p* values are 0.695, 0.787, and 0.725 at 1-s, 3-s, and 5-s, respectively.

**Fig 5:**
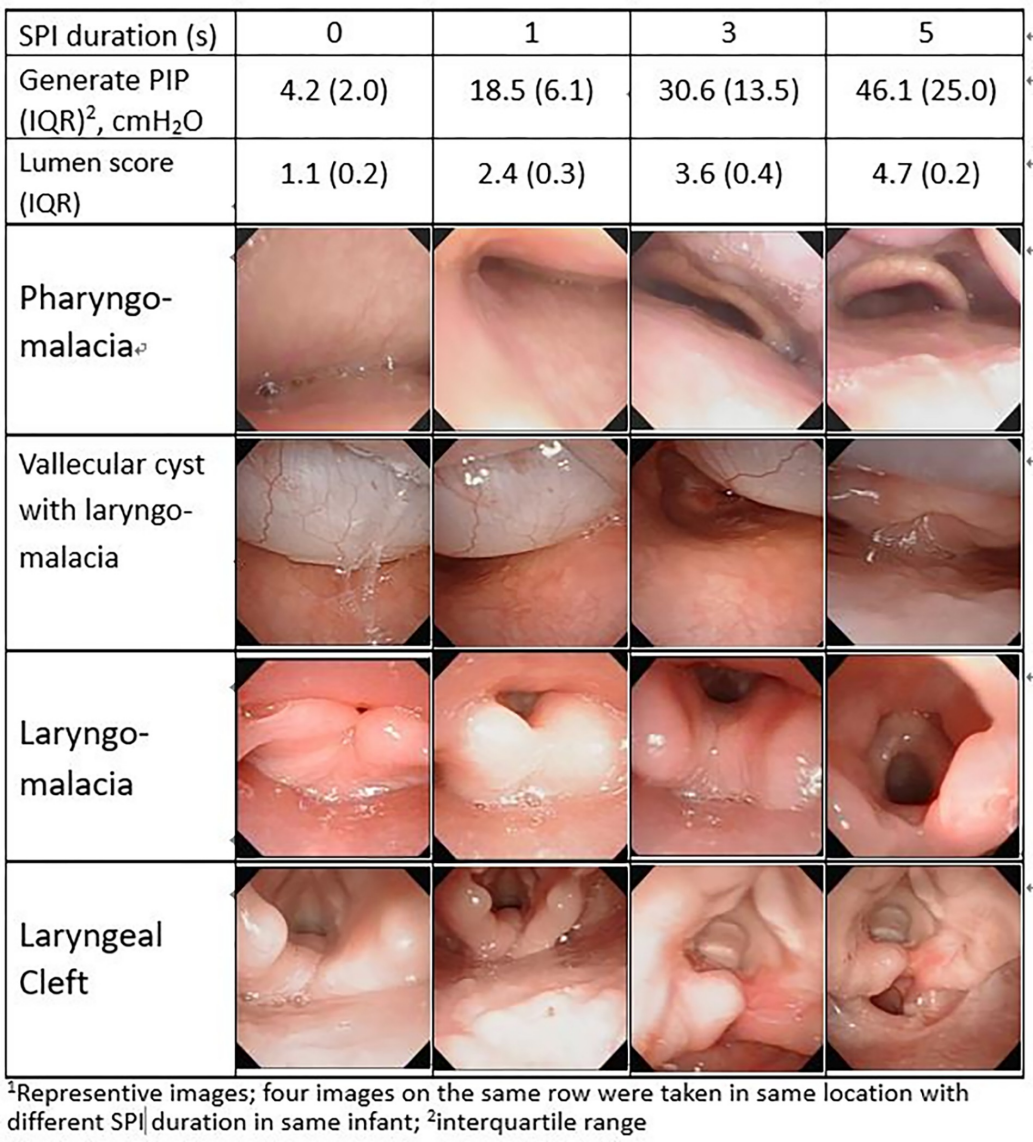
Changes of peak inflation pressure (PIP), lumen dimension score and associated image^a^ with four durations of sustained pharyngeal inflation (SPI) in pharyngeal space. The lumen spaces are gradually expand with increasing SPI durations and PIPs, which can disclose occlude lesions. Data shows in mean (interquartile range).

**Fig 6:**
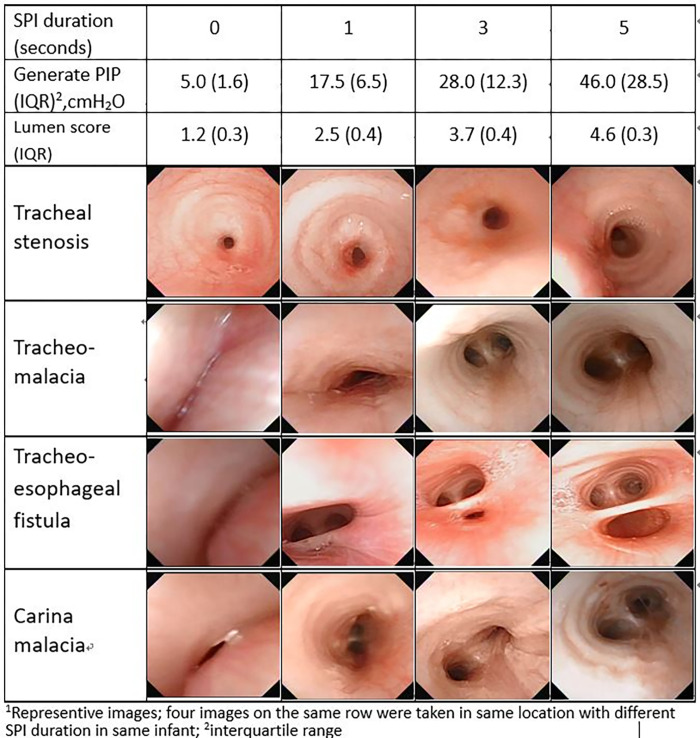
Changes of peak inflation pressure (PIP), lumen dimension score and associated image^a^ with four durations of sustained pharyngeal inflation (SPI) in tracheal lumen. The lumen spaces are gradually expand with increasing SPI durations and PIPs, which can disclose occlude lesions. Data shows in mean (interquartile range).

Figs 5 and 6 illustrate the corresponding levels of PIP, dimension scores, and associated images for each SPI duration in the pharyngeal space and the mid-tracheal lumen, respectively. On the same row of both Figs, all four images were taken at the same location of the same infant but at different SPI durations. These images all showed progressive and significantly expansion with increased durations of SPI [S2 and S3 Videos]. During the SPI-FB examinations, gradual and dynamic lumen expansions facilitated accurate and comprehensive visual evaluation of lesions, which were otherwise obscured with shorter NC durations but became obvious with longer durations of NC. This expansion effect was particularly significant for pressure-sensitive dynamic lesions, such as lumen malacia, vallecular cyst, hidden laryngomalacia [S4 Video], laryngeal cleft [S5 Video], and tracheal fistula [S6 Video], as shown in both Figs.

## Discussion

This is the first medical study involving this novel SPI-FB technique in pediatric patients’ both upper and lower airways. The pharyngolaryngeal space and tracheal lumen in small children are pressure-sensitive and dynamic structures. This study demonstrates that a “PhO_2_-NC” skill can act as a SPI technique, dynamically increasing the PIP, expanding the airway lumens, and therefore assisting accurate examination of FB in infants and small children. Compared to traditional NIV techniques, the PhO_2_-NC does not require additional equipment other than a soft catheter for continuous oxygen delivery.

FB examination through the nasal route to the entire reachable airway can offer real-time and dynamic visualization of intraluminal structures. In this study, gradually increasing the NC durations from 0 to 5s showed immediate expansion of all visible lumens, particularly in the malacia areas. Despite partial blockage by the bronchoscope itself in proximal pathway at the larynx, subglottis, and upper trachea, the measured PIPs at the mid-trachea are near identical to the pharynx at different SPI durations at 1 s, 3 s, and 5 s. This indicates that sufficient oxygen positive pressure ventilation (PPV) do transmit from the pharynx into the trachea which benefits subject’s oxygenation and ventilation during FB. This operator-controllable NC skill allows the FB to thoroughly and precisely evaluate intraluminal structures. As represented in Figs 5 and 6, these pressure-sensitive and dynamic lesions such as malacia, cyst, hidden laryngomalacia, cleft, and fistula could be clearly identified and assessed with appropriate SPI durations. Without dynamic and sufficient expansion of lumens, these lesions may remain undisclosed.

Physiologically, the SPI of continuous PhO_2_ and intermittent NC provide effects of “apneic oxygenation” and PPV, respectively. Multiple randomized controlled trials have shown that “apneic oxygenation” setting can significantly prolong periods of safe oxygen saturation and reduce the risk of hypoxemia in patients of apnea, under sedation, with muscle paralysis or difficult airways [19–23]. PhO_2_-NC offers an adjustable oxygen PPV which allows for diagnostic purposes and even various therapeutic FB interventions [8–17] such as laser ablation of vallecular cyst [10], laser laryngoplasty [10,11], balloon dilatation, stent implantation and repair [12–14], foreign body removal [15], and esophageal management [16,17]. In this study, the levels of generated PIP had briefly reached beyond 45 cmH_2_O at the end of 5-s NC, although without complications, it should be alert, carefully, and closely monitoring.

This SPI modality offers several clinical advantages;

1. It can easily execute with only a flexible (nasopharyngeal) catheter and a continuous oxygen flow. It is simple, feasible, and cost-effective in scenarios with limited resources.
2. Compared to other SPI devices, such as facemasks or nasal prongs, the gas-exchange interface bypasses the dropped tongue, closes to the larynx, and get less ventilation dead space.
3. During SPI-FB, the operator can perform NC and handle the bronchoscope simultaneously while providing oxygen PPV. This study demonstrates that SPI-FB with longer NC durations provides a more detailed evaluation of the airway and identifies lesions that may be inconspicuous without NC or shorter NC durations.
4. This PhO_2_-NC technique can provide oxygenation PPV before, during, and after airway interventions for subjects with hypoxia, apnea, compromised, or difficult airway. Therefore, it may serve as an alternative for resuscitative ventilation.

This SPI can be a double-edged sword: although it benefits to FB performance, the high PIPs are dangerous for air-leaks and other consequences such as inflammatory response phenomena like in Ventilator Induce Lung Injury [24,25]. In addition, high intrathoracic pressure reduces the venous return and impair the cardiac output and blood pressure. In clinical application of this SPI, excessive expansion of the airways, NC more than 3s (PIP more than 30 cmH_2_O) should be optional and transient (1–2 seconds) as needed.

There are some limitations in this study. It was a single-center study with a small number of enrolled cases. It is essential to conduct studies with more cases to prove its efficacy. Proficiency in performing this PhO_2_-NC technique might vary among operators. Training programs to improve the proficiency of operators to conduct PhO_2_-NC and SPI-FB are necessary before the wide application in clinical practice.

## Conclusion

Based on this study, we suggest that using PhO_2_-NC as a SPI technique with durations of up to 3s is relatively safe, simple, noninvasive, and valuable to provide sufficient PIP levels for detailed FB examination of the pharyngolaryngeal space and tracheal lumen. In selected children, prolonging SPI up to 5s should be cautious and carefully monitoring its risks.

## Supporting information

S1 Video Gross video of the technique “sustained pharyngeal inflation supports flexible bronchoscopy” in an infant. (MP4)

S2 Video Bronchoscopy view of sustained pharyngeal inflation in upper airway. (MP4)

S3 Video Bronchoscopy view of sustained pharyngeal inflation in lower airway. (MP4)

S4 Video Bronchoscopy view of sustained pharyngeal inflation in case of vallecular cyst with laryngomalacia. (MP4)

S5 Video Bronchoscopy view of sustained pharyngeal inflation in case of laryngeal cleft. (MP4)

S6 Video Bronchoscopy view of sustained pharyngeal inflation in case of tracheomalacia associated with tracheoesophageal fistula. (MP4)

S1 File Study IRB. (PDF)

S2 File “SPI-U-L-Aw study” data. (PDF)
