# Supplementary material for: Sustained pharyngeal inflation in infant airway—Flexible bronchoscopy measurements
Source: PLoS One. 2023 Nov 22;18(11):e0294029. doi: 10.1371/journal.pone.0294029 (PMC10664907; doi:10.1371/journal.pone.0294029)
Supplement: S1 File — (PDF) [file pone.0294029.s007.pdf]

中國醫藥大學附設醫院
CHINA MEDICAL UNIVERSITY HOSPITAL
台中市北區育德路2號
2 Yude Road, Taichung, 40447, Taiwan (R.O.C.)
TEL：886-4-22052121

中國醫藥大學暨附設醫院研究倫理委員會

Tel: 886-4-22052121 ext: 1925　Fax: 886-4-2207-1478　台中市北區育德路 2 號

臨床試驗/人體研究通過證明書

計畫名稱：幼兒施予持續充氣法時下氣道腔壓力與管腔變化—以軟式內視鏡監測
計畫編號/本會編號：　/ CMUH109-REC1-194
計畫主持人：兒童醫院胸腔科宋文舉主治醫師
執行機構：中國醫藥大學兒童醫院
通過日期：2021 年 01 月 26 日
計畫有效日期：2022 年 01 月 25 日
計畫書：Version 01, Date: Dec. 03, 2020
中文摘要：Version 01, Date: Dec. 03, 2020
英文摘要：Version 01, Date: Dec. 03, 2020
受試者同意書：Version 01, Date: Dec. 01, 2020
數據資料及安全監測計畫檢核表：Version 01 / 2020.12.06
持續審查頻次：每 12 個月一次

上述計畫已於 2021 年 01 月 06 日 經中國醫藥大學暨附設醫院研究倫理委員會第一審查委員會 2021 年第 01 次審查會議審查。本委員會的運作符合優良臨床試驗準則及國內相關法令。委員會決議此計畫之持續頻次如上述所示。請在持續審查必須進行前二個月向本會檢送完整之期中報告。

此計畫任何部分若經更改，必須在執行前重新提交本會審查及核准。此外，計畫主持人必須依時通報嚴重不良事件及涉及受試者或其他人風險的非預期問題。

中國醫藥大學暨附設醫院 研究倫理委員會 台中市北區育德路2號

主任委員 [illegible]

中　華　民　國　一　一　〇　年　二　月　一　日

CMUH

中國醫藥大學附設醫院
CHINA MEDICAL UNIVERSITY HOSPITAL
台中市北區育德路2號
2 Yude Road, Taichung, 40447, Taiwan (R.O.C.)
TEL : 886-4-22052121

Research Ethics Committee
China Medical University & Hospital, Taichung, Taiwan

Tel: 886-4-22052121 ext: 1925 Fax: 886-4-2207-1478

Clinical Trial/Human Research Approval

Date：Feb. 01, 2021

Protocol Title：Sustained inflation effects on lower airway pressure and lumen changes in infant—flexible endoscopy measurement
Protocol No. / CMUH REC No.： / CMUH109-REC1-194
Name of Principal Investigator：Wen-Jue Soong (Attending Physician, Pediatric Pulmonology)
Name of Institution：China Medical University Children's Hospital
Date of Approval：Jan. 26, 2021
Date of Expiration：Jan. 25, 2022
Protocol：Version 01, Date: Dec. 03, 2020
Chinese Synopsis：Version 01, Date: Dec. 03, 2020
English Synopsis：Version 01, Date: Dec. 03, 2020
Informed Consent Form：Version 01, Date: Dec. 01, 2020
DSMP：Version 01 / 2020.12.06
Frequency of Continuing Review：once per every 12 months

This is to certify that the above referenced research project has been reviewed by the 2021 1st meeting of the Research Ethics Committee (REC) I of the China Medical University and Hospital on Jan. 06, 2021. The REC is organized under, and operates in accordance with, the Good Clinical Practices guidelines and the governmental laws and regulations. The frequency of continuing review for the research project determined by the REC is mentioned above. Please submit a completed progress report at least two months before the time at which continuing review must occur.

All the amendments to the research project should be re-submitted and approved by the REC BEFORE implementation. Also, the principal investigator is required to report all serious adverse events and unanticipated problems involving risks to the subjects or others on time.

Martin M-T Fuh MD, DMSci.
Chairman, Research Ethics Committee I
China Medical University & Hospital

CHINA MEDICAL UNIVERSITY & HOSPITAL
The Research Ethics Committee
No. 2 Yude Road, Taichung, Taiwan, R.O.C.
