# Supplementary material for: Sustained pharyngeal inflation in infant airway—Flexible bronchoscopy measurements
Source: PLoS One. 2023 Nov 22;18(11):e0294029. doi: 10.1371/journal.pone.0294029 (PMC10664907; doi:10.1371/journal.pone.0294029)
Supplement: S2 File — (PDF) [file pone.0294029.s008.pdf]

De-identified study data (SPI-PIP-bronchoscopy measurement)

Case No.	Age	Gender	Weight (Kg)	High flow (L)	Upper Aw 0 s (cmH_2O)	Upper Aw 1 s (cmH_2O)	Upper Aw 3 s (cmH_2O)	Upper Aw 5 s (cmH_2O)	Lower Aw 0 s (cmH_2O)	Lower Aw 1 s (cmH_2O)	Lower Aw 3 s (cmH_2O)	Lower Aw 5 s (cmH_2O)
Case-01	36M	Male	13.5	10	0	10.8	22.3	27.6	3.2	15.7	22.6	26
					0	14.2	23.8	24.6	6.2	18.9	21.8	30
					0	12.8	18.6	27.8	5.8	18.9	21.9	22.8
Case-02	12M	Female	10	10	3.2	12.8	36.8	76.2	5.2	18.6	38.9	75.2
					3.2	21.8	41.9	64.8	3.2	12.8	39.2	82.6
					4.2	26.4	33.9	63.8	4.8	17.8	35.6	49.8
Case-03	6M	Male	7.3	7	3.2	22.8	34.6	44.8	0	26.4	54.5	83.6
					4.6	24.8	42.4	77.9	5.4	16.8	33.2	65.8
					3.4	19.6	32.8	70.2	4.3	21.8	39.6	73.5
									0	14.8	40.5	66.8
Case-04	7M	Male	7	7	5.1	14	21.9	33.4	6.9	8.3	10.1	30.6
					0	21.9	35.6	45.8	6	25.8	32.3	72.6
					6	20.2	38.9	50.8	9.8	16.9	38.4	50.8
									14	25.7	53.4	63.8
Case-05	23M	Male	5	4	3.4	21.8	53.2	79.6	3.2	22.5	43.2	64.8
					7	19.8	39.6	81.2	7.2	22.5	26.8	63.4
					0	23.5	50.1	70.2	5.2	18.1	30.6	64.7
Case-06	3M	Female	5	5	0	17.8	34.5	56.4	4.8	13.2	34.1	54.5
					3.5	24.4	48.6	57.1	5.1	24.8	43.8	63.4
					0	20.2	41.5	65.8	4.1	13.5	34.8	77.9
Case-07	12M	Male	11	10	0	16.8	19.5	30.2	4.5	16.9	21.4	26.8
					5.8	16.9	20.5	38.5	5.2	20.3	33.5	50.1
					4.8	22.5	33.5	44.6	4.2	17.8	27.4	56.8
Case-08	36M	Female	14	10	3.3	17.1	26.2	44.8	6.1	15.8	23.7	30.6
					5.1	19.6	22.1	51.2	8.2	17.8	28.7	35.6
					5.2	21.2	34.5	57.8	3.8	20.1	31.2	34.6
Case-09	12M	Female	7.6	7	4.7	19.1	24.5	64.2	0	19.8	32.4	80.1
					0	23.5	31.2	61.8	3.3	26.1	38.5	83.2
					3.1	25.6	33.1	62.4	5.1	29.4	39.6	72.8
					4.4	25.5	40.1	62.8	4.4	26.5	39.8	87.6
									4.6	22.3	32.5	61.5

Case-10	5M	Male	6	6	0	13.8	27.9	32.6	0	22.1	30.2	40.8
					0	19.8	31.7	56.8	6.1	22.0	32.8	50.2
					0	22.4	35.1	56.2	0	22.5	35.4	50.8
Case-11	6M	Female	5.05	5	0	13.2	18.9	32.3	4.4	16.9	25.1	36.7
					3.2	18.7	24.8	37.8	4.1	15.8	27.6	44.8
					0	22.2	23.8	42.2	3.8	15.9	24.5	39.8
Case-12	3M	Male	5	5	0	9.2	19.5	26.2	6.2	8.3	20.1	27.4
					0	16.2	21.2	31.7	5.4	13.1	17.5	27.8
					0	5.8	23.2	31.2	5.6	19.8	27.2	33.5
Case-13	36M	Male	14.2	10	3.2	13.8	26.4	49.8	7.2	12.8	26.7	52.1
					4.6	14.5	27.6	39.4	3	16.7	26.2	52.8
					5.4	13.4	35.8	55.4	5.1	17.5	24.5	49.2
Case-14	3M	Male	5	6	4.2	20.1	50.2	66.1	10	20	34.8	50.2
					8.1	22.8	54.8	70.0	5.8	22.4	33.9	53.8
					11	28.1	34.8	65.1	6.8	22.5	38.4	59.6
Case-15	6M	Female	5	5	0	16.4	21.2	34.8	3.5	11.4	17.2	25.7
					0	19.8	36.8	51.2	0	10.8	14.2	18.5
					0	15.4	35.2	45.8	0	11.4	15.4	19.7
									6.4	13.8	24.2	30.2
Case-16	12M	Male	10.5	8	5.4	20.2	35.6	47.4	6.8	25.1	29.6	37.8
					5.6	16.9	30.2	61.3	8	30.1	38.9	53.2
					4.8	18.1	37.6	44.8	5.1	27.6	34.9	57.2
Case-17	1M	Male	5.05	5	5.1	10.2	38.1	39.8	4.8	22.1	25.8	27.9
					4.5	19.3	33.2	37.8	0	12.7	34.2	41
					4.2	15.4	26.7	35.7	0	13.7	24.1	39
					3.2	15.2	22.3	29.8				
Case-18	4M	Female	5.5	5	4.5	12.7	16.9	29.4	3.4	10.2	19.4	38.4
					5.2	13.9	22.7	30.4	0	12.4	23.5	29.8
					0	12.3	18.4	28.5	3.2	13.1	19.4	31.3
									0	10.6	21.2	33.5
Case-19	11M	Female	7.2	7	4.2	8.6	18.9	29.4	3.5	10.2	19.1	31.3
					3.2	16.4	33.1	43.4	3.6	12.3	24.8	33.5
					3.8	16.8	26.5	38.2	3.9	13.5	22.7	33.5
Case-20	6M	Male	7.6	7	0	13.4	21.3	37.2	9.8	12.7	19.6	30.2
					4.6	12.4	22.6	43.2	0	11.8	32.7	30.5
					6.7	22.1	24.8	49.6	0	14.3	26.7	42.8
